# How We Can Change Clinical Practice Using Antioxidant Molecules?

**DOI:** 10.3390/antiox11061116

**Published:** 2022-06-04

**Authors:** Gaia Favero, Rita Rezzani

**Affiliations:** 1 Anatomy and Physiopathology Division, Department of Clinical and Experimental Sciences, University of Brescia, 25123 Brescia, Italy; rita.rezzani@unibs.it; 2 Interdipartimental University Center of Research “Adaption and Regeneration of Tissues and Organs (ARTO)”, University of Brescia, 25123 Brescia, Italy; 3 Italian Society of Orofacial Pain (SISDO), 25123 Brescia, Italy

The collection of studies in this Special Issue, “*The Role of Antioxidant Molecules and Melatonin in Cellular Protection*”, published in Antioxidants (accessed on 30 April 2021; https://www.mdpi.com/journal/antioxidants/special_issues/antioxidant_melatonin), would prefer to highlight the current knowledge and presents new contributions on oxidative stress-dependent signaling pathways in various pathophysiological conditions as well as new antioxidant strategies to prevent/treat the pathological status. This Special Issue comprises 5 research studies and 9 review articles providing important contributions to the Special Issue topic by distinguished experts.

The original study performed by Szeiffova Bacova et al. [1] explore the anti-arrhythmic potential of melatonin and omega-3 showing that both compounds were powerful to attenuate abnormal connexin-43 topology along with the suppression of collagen deposition in catecholamine-stressed heart of normotensive and hypertensive. The Authors emphasized that both antioxidant compounds blunted acute and chronic pro-arrhythmic signaling and related ventricular fibrillation.

To gain insight into the antioxidant role(s) and regulatory mechanism(s) of melatonin in yeast, Sunyer-Figueres [2] presented, for the first time, the yeast transcriptional response to exogenous melatonin. The Authors observed that melatonin crossed cellular membranes and reprogrammed the cellular machinery to achieve tolerance to oxidative stress.

Lomovsky et al. [3] investigated the combined effect of melatonin and navitoclax on a cell model of acute promyelocytic leukemia. Melatonin and navitoclax dropped membrane potential, increased oxidative reactive oxygen species production, modulate endoplasmic reticulum stress, enhance autophagy and apoptotic signaling cascade. This study so suggests that melatonin in combination with anti-tumor compounds has important anti-neoplastic effect(s).

The study of Ali et al. [4] identified for the first time the protective roles of melatonin in pepper plants observing that exogenous melatonin is a promising approach against plant infestation. The Authors reported that melatonin treatment kept the plant concentration of intracellular hydrogen peroxide at a steady-state level and increased the activities of endogenous antioxidant enzymes. In addition to induce plant innate immunity against pathogens, melatonin application attenuated destructive pathogen growth by inhibiting mycelia extension.

Using an animal model of diabetic nephropathy, Hsiao et al. [5] investigated the therapeutic effect(s) of melatonin combined with extracorporeal shock wave therapy and reported that this approach ameliorates kidney morphology reducing oxidative stress, inflammation, apoptosis and glomerular fibrosis.

Excellent review studies are also included in this Special Issue, emphasizing both the importance of oxidative stress in various chronic diseases and the impact of antioxidant molecules in fighting cellular injury.

Sadanandan et al. [6] summarized the current preclinical and clinical studies regarding melatonin-based therapeutics to rescue the central nervous system following ischemia. Notably, recent clinical trials exploring the curative potential of melatonin in stroke and neurodegenerative diseases display that melatonin, acting as an antioxidant and free radical scavenger, ameliorates neuroinflammation and promotes brain tissue restoration.

The review manuscript of Ikram et al. [7] reported the studies conducted on the role of caffeine in the management of different neurological disorders. Caffeine has shown strong antioxidant and anti-inflammatory effects and so it may provide symptomatic relief against devastating neurodegenerative conditions.

Ferlazzo et al. [8] reviewed the main biological activities and effects of melatonin and its metabolites in treating oxidative stress- and/or inflammation-related disorders. The Authors also reported the actual knowledge on the combination of melatonin with traditional therapies in increasing the efficiency of the treatment for infective disorders, cardiovascular diseases, cancers and neurodegenerative disorders.

In a systematic review, Sumsuzzman et al. [9] collected and discussed the actual studies on melatonin clinical applicability in the treatment of burn-induced distant-organ injury. Meta-analysis indicates that melatonin has significant therapeutic potential by regulating oxidative-stress and the inflammatory responses.

Félix et al. [10] summarized in a review article the several direct and indirect approaches to reduce the spermatozoa oxidative processes reporting also the factors affecting the efficiency of antioxidant molecules and their mechanisms of action. Special attention was given to the melatonin pathway and its potential activity in preventing oxidative stress damage on fish spermatozoa.

Jaworek et al. [11] systematically presented experimental and clinical data that investigated the role of melatonin against the atopic dermatitis. The Authors concluded that melatonin has powerful antioxidant effects and its implication in the defense of inflammatory reaction of atopic dermatitis patients is very likely.

Langston-Cox et al. [12] conducted a review on the preclinical and clinical evidence for melatonin as a possible adjuvant therapy for preeclampsia. The antioxidant melatonin effects have been elucidated in placenta cells, in mitochondria and in vascular cells, all key contributors to the pathogenesis of preeclampsia.

Pardo-Hernández et al. [13] summarize the most recent research conducted on metal toxicity in plants and reported how exogenous application of melatonin may improve the negative effects of metal stress in plants.

The review article of Favero et al. [14], Editor of this Special Issue, reported in vitro, in vivo, and clinical studies on the state of the art of polyphenols application against dry eye disease (DED) and discussed that these natural compounds present promising outcomes against oxidative stress-related pathogenic status. Current treatment involves reducing environmental causes, discontinuing medications that induce or worsen DED and managing contributing ocular or systemic conditions [15,16]. Lubrication with tear substitutes is a mainstay of DED treatment; however, very few randomized controlled trials have compared interproducts superiority. Furthermore, ocular lubricants do not target the underlying pathophysiological DED features and the mechanisms of any palliative actions are generally poorly understood. Therefore, at the moment, there are no resolving treatments and DED patients continue to suffer symptoms and report dissatisfaction with the prescribed treatment. Significant studies have been carried out in the past decade to develop ocular therapeutics and the research is ongoing to find complementary/innovative treatments against DED. Polyphenols show potential therapeutic effects in numerous pathologies [17,18,19], but despite various supportive studies suggesting positive effects of polyphenols against DED [14,20], their clinical use is still questioned and limited. Why? A possible reason is the lack of solid clinical trials demonstrating polyphenols therapeutic benefits. Polyphenols have shown significant potential against DED in many preclinical studies, but not enough clinical trials are actually performed [21,22]. It is fundamental to proceed with other clinical trials taking into account, to avoid the same trouble, the omega-3 results of the Dry Eye Assessment and Management (DREAM) study that stated “… no evidence of a beneficial effect of omega-3 supplements on the symptoms and signs DED relative to placebo supplements”. This sentence seemed very surprising to many clinics who have recommended omega-3 supplements to their patients for years with evident success. This trouble may be related, as reported by Asbell [23], to controversy over the data interpretation (some based on the facts and some based on inaccurate assumptions or misstatements). There are, in fact, several factors that need to be considered when will be design and execute a randomized clinical trial. In particular, future clinical trials involving DED patients have to take care of low polyphenols bioavailability, the complexity of the DED pathophysiology and patient’s compliance and perception of treatment effectiveness. Another major challenge with the state of the current evidence for DED treatment is that most clinical trials have to focused on demonstrating improvements in short-term outcomes, as needed for regulatory approval, but DED is a chronic ocular surface condition.

The Authors in the present Special Issue wants to emphasize that exogenous antioxidants have been recognized as one of the most promising therapeutic options toward the precautions and treatments of many oxidative stress-related diseases. In fact, over the past decade, several kinds of antioxidants have been evaluated for antioxidant therapy showing great effects. However, persist concerns regarding the inherent instability, limited free radical scavenging ability, nonspecific distribution and short cycle half-life limit or avoid their clinical application.

With these limitations in mind, there are many opportunities for clinicians and researchers to conduct studies that would add a key contribution to the body of knowledge and would help the future management of several oxidative stress relevant diseases. This Special Issue will be helpful to Researchers in the development of innovative preventive or therapeutic strategies that may lead to a clinical translation soon.

The Guest Editor wants to thank all the Authors and the Reviewers who contributed to the success of this Special Issue and the Antioxidants team for their valuable and constant support.
